# The role of iron metabolism in the pathogenesis and treatment of multiple sclerosis

**DOI:** 10.3389/fimmu.2023.1137635

**Published:** 2023-03-17

**Authors:** Eduardo Duarte-Silva, Sven G. Meuth, Christina Alves Peixoto

**Affiliations:** ^1^ Center for Research in Inflammatory Diseases (CRID), Ribeirão Preto Medical School, Department of Pharmacology, University of São Paulo, Ribeirão Preto, SP, Brazil; ^2^ Network of Immunity in Infection, Malignancy and Autoimmunity (NIIMA), Universal Scientific Education and Research Network (USERN), Ribeirão Preto, SP, Brazil; ^3^ Department of Neurology, Medical Faculty, University Hospital Düsseldorf, Düsseldorf, Germany; ^4^ Laboratory of Ultrastructure, Aggeu Magalhães Institute (IAM), Recife, PE, Brazil; ^5^ National Institute of Science and Technology on Neuroimmunomodulation (INCT-NIM), Oswaldo Cruz Institute, Oswaldo Cruz Foundation, Rio de Janeiro, Brazil

**Keywords:** experimental autoimmune encephalomyelitis (EAE), multiple sclerosis (MS), iron, iron metabolism, ferroptosis, pathogenic T lymphocytes

## Abstract

Multiple sclerosis is a severe demyelinating disease mediated by cells of the innate and adaptive immune system, especially pathogenic T lymphocytes that produce the pro-inflammatory cytokine granulocyte-macrophage colony stimulating factor (GM-CSF). Although the factors and molecules that drive the genesis of these cells are not completely known, some were discovered and shown to promote the development of such cells, such as dietary factors. In this regard, iron, the most abundant chemical element on Earth, has been implicated in the development of pathogenic T lymphocytes and in MS development *via* its effects on neurons and glia. Therefore, the aim of this paper is to revise the state-of-art regarding the role of iron metabolism in cells of key importance to MS pathophysiology, such as pathogenic CD4^+^ T cells and CNS resident cells. Harnessing the knowledge of iron metabolism may aid in the discovery of new molecular targets and in the development of new drugs that tackle MS and other diseases that share similar pathophysiology.

## 1 Introduction

Multiple Sclerosis (MS) is a debilitating and autoimmune disease affecting the central nervous system (CNS) that is classically characterized by demyelination and neuroaxonal degeneration (1). From the immunological standpoint, it is a complex disease and many innate and adaptive immune cells play essential roles driving disease progression, such as T and B lymphocytes and myeloid cells. Importantly, pathogenic T cells play an essential role in disease progression *via* effector mechanisms that include licensing myeloid cells that infiltrate the CNS to cause and perpetuate damage (2). Apart from this, other pathological processes take place in the CNS during MS, such as dysregulation of the blood-brain barrier (BBB) and of the coagulation system, defects in remyelination, gut microbiome alterations, changes in cellular metabolism and in microbial metabolites, dysfunction of the adenosinergic signaling pathway, increased apoptosis and defective autophagy (3–7).

Iron, the most abundant chemical element on Earth, plays important roles in biological processes that are fundamental to the maintenance of host homeostasis, such as proper ATP generation and immune system modulation. However, iron dyshomeostasis has been implicated in the development of pathogenic T lymphocytes. Therefore, the aim of this paper is to present the start-of-art regarding the role of iron metabolism in the function of relevant cells subtypes that are of utmost importance to the development of MS, such as CD4^+^ T cells and CNS resident cells. Furthermore, we highlight how iron metabolism could be used therapeutically to tackle MS. Since some cell types and signaling pathways are shared by many autoimmune, neuroinflammatory and neurodegenerative diseases, the topics of the current paper may also be of relevance to other diseases not covered here.

## 2 Autoimmunity and pathogenic lymphocytes

Autoimmune diseases are characterized by a break in central and peripheral tolerance to self, resulting in the immune system attacking its own host (8). It is currently estimated that this class of diseases affects 5%-8% of the people worldwide (9), which represents a global burden that is often associated with severe disabilities and a high socioeconomic cost. Although the key players underpinning these disorders are T lymphocytes, it is now recognized that autoimmunity can also develop in an antigen-independent manner (10). It is widely accepted that autoimmune disorders arise as a result of a loss of balance between regulatory T (Treg) cells and Th17 cells (11) and restoring this balance is at the heart of several therapies. In addition to immune dyshomeostasis, environmental factors, such as lifestyle and diet, have been implicated in the pathogenesis of such diseases (12). Furthermore, genetic factors have also been associated with the development of autoimmunity. In this regard, mutations in specific genes, such as *il23r*, *ctla4*, *AIRE* and *FOXP3* have been well documented (13).

CD4^+^ T helper (Th) lymphocytes can be broadly divided into different categories, depending on the effector cytokines they secrete and the transcription factors (TFs) that are involved in their differentiation. For instance, Th1 cells are characterized by the production of IFN-γ and the expression of the TF T-bet, playing an important role in the defense against intracellular pathogens. Another example includes Th17 cells, which are characterized by the expression of the master transcription factor RORγt and the secretion of IL-17A, IL-17F, IL-22 and IL-10 (often called homeostatic of non-pathogenic Th17 cells) (14). Although these cells play an important role in the defense against extracellular pathogens and in the modulation of epithelial barrier function, they can become pathogenic when exposed, for instance, to a combination of IL-1β, IL-6 and IL-23, which activates the pathogenic signature of these cells. Of note, T cell receptor (TCR) activation leads to chromatin remodeling and recruitment of pioneer TFs such as IRF4 and BATF to bind to specific chromatin regions. In the context of inflammation, IL-6 binding to IL-6R triggers the activation of JAK signaling pathway, enabling STAT3 to translocate to the nucleus and bind to the open chromatin. Together with BATF, IRF4 and the co-activator p300, STAT3 promotes the transcription of the gene RORγt gene (*rorc*). By its turn, RORγt is now able to bind to chromatin and promote the expression of Th17 specific genes (15). It should be noticed that signaling through IL-6R, IL-21R and IL-23R leads to STAT3 activation and RORγt expression, which on the one hand triggers the production of IL-17 and, on the other hand, inhibits the expression of the TF Foxp3, important for the differentiation of Treg cells (16). It is important to highlight that, apart from the effector cytokines, these T lymphocytes are also characterized by their unique transcriptional signature. For instance, non-pathogenic Th17 cells can express *Ahr*, M*af, Il10* and *Il9*. Conversely, pathogenic Th17 cells express *Tbx21, Ifng, Il23r, Gzmb* and *Csf2* (14).

As a consequence of being exposed to the aforementioned cytokines, these T lymphocytes can produce IL-17A, IL17F, IL-21, IL-22 (12, 17), which exert immunomodulatory effects in different target-organs and target cells, contributing to homeostasis and autoimmunity. For instance, it is known that IL-17 and IL-22 secreted by Th17 cells cause increased permeability in the blood-brain-barrier (BBB), which allows them to migrate into the brain parenchyma and initiate disease (18). Moreover, reconstitution of IL-17 expression in intestinal epithelial cells restored susceptibility to develop CNS autoimmunity in mice (19). In this regard, harnessing the knowledge of TFs and signaling pathways that govern the differentiation of these pathogenic cells may unravel novel molecular targets that may aid in the development of new drugs to tackle autoimmune disorders.

One important “subtype” of lymphocytes are T helper (Th) cells that produce granulocyte-macrophage colony-stimulating factor (GM-CSF), initially hypothesized to be a growth factor for cells of the myeloid lineage. However, recent research has shown that GM-CSF is a proinflammatory cytokine (2, 20–22). To date, accumulating evidence points to the fact that GM-CSF acts on myeloid cells that infiltrate the CNS, licensing them to promote and perpetuate neuroinflammation by increasing their potential to phagocytose, produce ROS and secrete pro-inflammatory cytokines (PICs) (2). Of note, many cell types can be the source of GM-CSF during CNS autoimmunity, such as CD8^+^ T cells, innate lymphoid cells (ILCs), NK cells, γδ T cells, but CD4^+^ T lymphocytes were described as the main producers of this cytokine (20). Of note, due to the phenotypic plasticity of Th17 cells and the stimuli present in the microenvironment, they can transdifferentiate into Th1-like cells (ex-Th17 cells), interrupt the production of IL-17, but express IFN-γ and GM-CSF at the same time (23). Of note, although some groups consider GM-CSF producers a different T cell subtype (often called ThGM cells) (24), others are more cautious and propose that the expression of this cytokine is only part of a program that characterizes more pathogenic T lymphocytes (14). Giving the importance of such cells to inflammation and autoimmunity, gaining knowledge of the metabolic pathways that drive their development and expansion is key for the development of efficacious therapies to tackle inflammatory and autoimmune disorders. In this regard, the iron metabolism is emerging as a central hub that regulates not only energy metabolism, but also immunity.

## 3 Iron metabolism and encephalitogenic T lymphocytes in MS: A therapeutically promising duo

**Figure 1:**
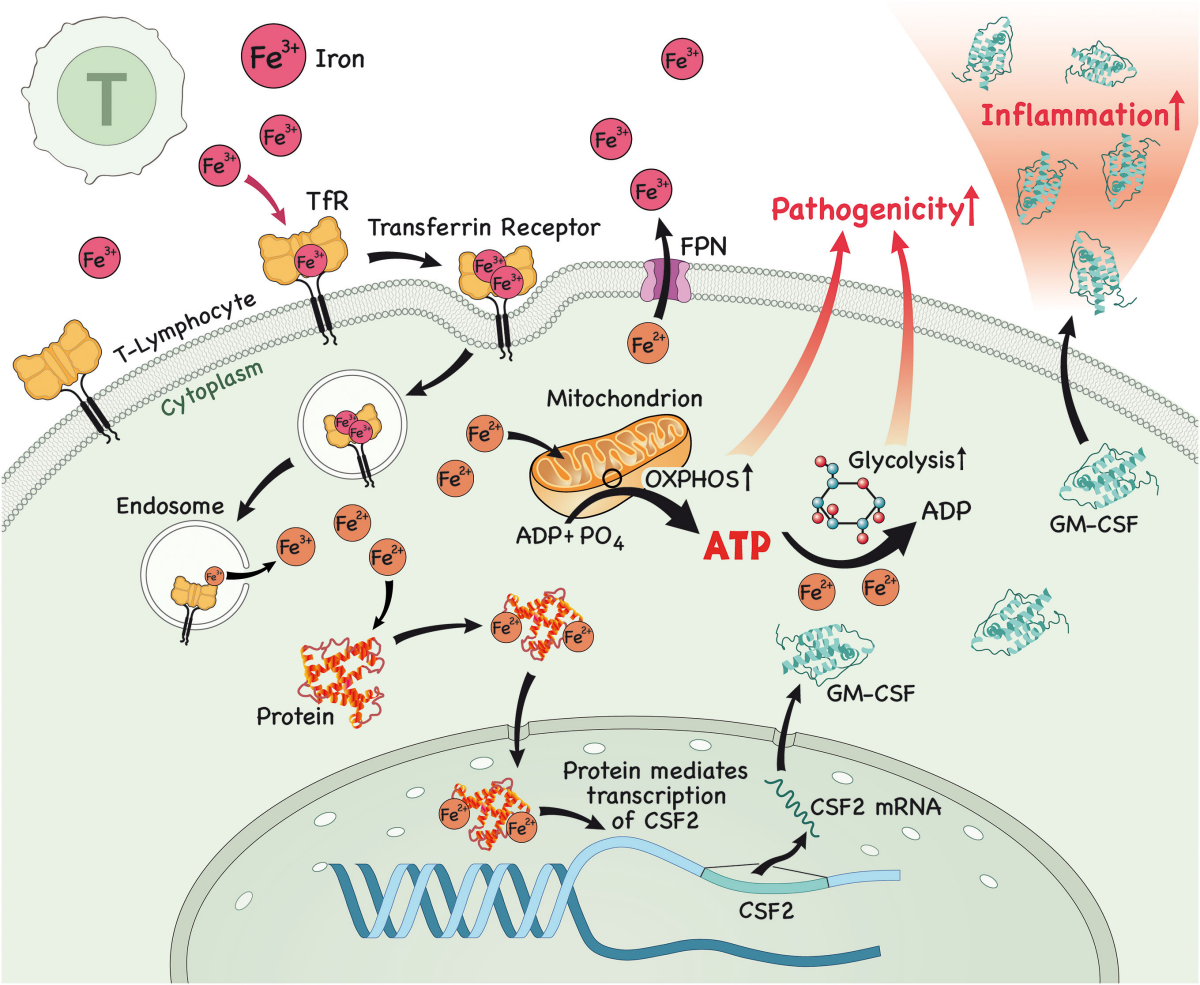
In the lymph node (LN), iron is imported into T lymphocytes. Once in the cytoplasm, iron interacts with proteins and this complex then translocates to the nucleus to modulate the expression of *csf2*, which encodes for the protein GM-CSF. After protein translation, GM-CSF is secreted by these pathogenic T lymphocytes, causing inflammation. Alternatively, iron triggers glycolysis and OXPHOS in T lymphocytes, which enables them to acquire a more pathogenic profile, further contributing to inflammation.

Iron (Fe) is the most abundant chemical element on earth and it can exist in two main forms: ferrous iron (Fe^2+^) or ferric iron (Fe^3+^). In the context of biological systems, iron is important because it takes part in the composition of metalloproteins or Fe-S clusters, which are key elements in oxygen transport (hemoglobin), electron transport chain (ECT), energy production (cytochrome C), oxygen storage (myoglobin) and host defense (NAPH oxidases [NOXs]), which influence immunity, central nervous system (CNS) physiology and behavior (25). Iron enters the body through its absorption in the intestine and then reaches the bloodstream *via* the export protein ferroportin (FPN). Once in the bloodstream, Fe^2+^ is oxidized by ferroxidases (such as ceruloplasmin [Cp] and hephaestin), enabling Fe^3+^ to bind to its transporter, transferrin (Trf), which transports iron throughout the body (25, 26). When the complex Fe^3+^-Trf reaches a target-tissue, it binds to transferrin receptor (TrfR or CD71), which is followed by endocytosis. Inside the endosome and due to the low pH and ferrireductases (such as six transmembrane epithelial antigen of the prostate 1 [STEAP-1]), Fe^3+^ dissociates from the complex and becomes Fe^2+^, which leaves the endosome through the transporter divalent metal transporter 1 (DMT1) (**Figure 1**). Inside the cell cytoplasm Fe^2+^ can have the following fates (26) (1): be stored in the form of Fe^3+^ in the protein ferritin, being unavailable to be used by the cell; (2) enter the mitochondria through the transporter mitoferrin, which plays a fundamental role in the formation of Fe-S clusters; (3) trigger signal transduction by binding to iron regulatory proteins (IRPs), which translocate to the nucleus and bind to iron responsive elements (IREs), allowing the modulation of genes important in iron metabolism, such as the gene encoding for TrfR and DMT1; (4) leave the cell trough the exporter FPN (26). Although iron is essential in cell physiology, excess iron in the form of Fe^2+^ is a potent source of oxidative damage, because Fe^2+^ reacts with hydrogen peroxide (H_2_O_2_), which generates Fe^3+^ and hydroxyl radicals (OH^.^). Together with other reactive oxygen species (ROS), OH^.^ can damage many cellular constituents, such as lipids, protein and nucleic acids. As a consequence, this triggers the generation of lipid peroxides, such as malondialdehyde (MDA) and 4-hydroxynonenal (4-HNE), which are extremely toxic to the cell because they initiate a newly characterized form of cell death termed ferroptosis (27), which is a key phenomenon in the pathogenesis of neurodegenerative diseases, such as Parkinson’s disease (PD) (28). Of note, during the course of EAE ferroptosis is heavily active and drives disease progression (29). On the other hand, pharmacological inhibition of ferroptosis by liproxstatin-1 (Lip-1) resulted in lessened disease course due to reduced infiltration of IFN-γ and IL-17A producing cells into the CNS (29). Additionally, administration of Cp led to reduced disease burden due to reduced accumulation of iron and a decreased in the levels of MDA as well as reduced neuronal death and infiltration of CD4^+^ T cells in the CNS (29).

Research has shown that iron has a fundamental role in the metabolism of innate and adaptive immune cells, specifically CD4^+^ T lymphocytes. It is now recognized that iron is able to modulate the differentiation of naïve CD4^+^ T cells to a more inflammatory or pathogenic phenotype, which has been associated with the genesis of inflammatory and/or autoimmune diseases affecting various organs, such as the intestine (colitis), skin (psoriasis) and CNS (MS). Unsurprisingly, it has also been demonstrated that GM-CSF production by CD4^+^ T lymphocytes occurs in an iron-dependent manner. It was shown that iron in the form of Fe^2+^ binds and stabilizes an RNA-binding protein known as PCBP1, which further stabilizes *csf2* mRNA (encoding for GM-CSF) (30). Furthermore, blocking iron entry in CD4^+^ T cells using an antibody against CD71 attenuated the differentiation towards a pathogenic phenotype and lessened disease severity in an animal model of MS, experimental autoimmune encephalomyelitis (EAE) (31). Is it important to highlight that GM-CSF production in T lymphocytes is driven by IL-23, which is secreted by antigen presenting cells (APCs) (32), binds to IL-23R and enables GM-CSF secretion by T cells (33–35), highlighting the importance of the iron/IL-17/IL-23/GM-CSF axis in the pathogenesis of autoimmune disorders.

Iron can also lead to the development of pathogenic T lymphocytes by another mechanism. A recent study has shown that iron is required to drive glycolysis in CD4+ T cells and in pathogenic T cells in the context of autoimmune myopathy (36). Pathogenic cells were shown to have higher PFKFB4 and CD71 expression, higher expression of glycolysis-related genes and higher glycolytic rate and oxidative phosphorylation (OXPHOS) in comparison to lymphocytes of healthy individuals (36). Interestingly, iron chelation with deferasirox (DFX) downregulated glycolysis and OXPHOS, glycolysis-related genes, such as *glut3* (36) and the TF HIF-1α, both required for proper pathogenic Th17 function (37–39), in CD4^+^ T cells. Of note, these effects were reversed when iron conjugated to transferrin was administered. Glucose uptake by healthy CD4+ T cells was also reduced after the administration of various iron chelators, such as ciclopirox (CPX), DFX and deferoxamine (DFO) (36). These effects were also observed when iron chelator was administered to pathogenic T cells, in addition to a downregulation of molecules involved in the glucose metabolism pathway, such as AKT, mTOR and PFKFB4, and of CD71 (36).

## 4 Iron and regulatory T cells: What is on the horizon?

The balance of Treg cells and T effector cells (Teffs), notably Th1 and Th17 cells, is fundamental to keep body homeostasis and prevent the development of an excessive immune response against self and non-self (antigens derived from diet or the commensal microbiota or even allergens (40, 41). Treg cells can often be subdivided into two types: thymus derived Treg cells (tTreg cells) and peripherally derived Treg cells (pTreg cells), both having their identity controlled by the master TF Foxp3 (40–43), although their differentiation and function appear to differ in several aspects (41). Treg cells can exert their suppressive function either by cell-cell interactions *via* molecules located on the cell membrane, such as CTLA-4, CD25, CD39, CD73 or by the secretion of soluble factors, such as TGF- β and IL-35 (41).

In MS, although Treg cell number is reported not to be changed, its suppressive function suppressive function is altered (44). However, the role of iron metabolism in Treg cells is still obscure. To date, it is suggested that iron overload induces an increased in the frequency of Foxp3^+^ CD4^+^ T cells *in vivo* (45), but the underpinning mechanisms still remain unidentified. Furthermore, iron chelators such as CPX, DFX and DFO were shown to increase the frequency of Foxp3^+^ CD4^+^ T cells *in vitro*, but not under in vitro-induced Treg (iTreg) cell differentiation (36). Notably, iron chelation was shown to increase the frequency of Foxp3^+^ CD4^+^ T cells *in vivo* in a model of autoimmune myopathy (36). More research is urgently needed to address unanswered questions regarding the immunomodulatory effects of iron in Treg cells and its implications in the context of neuroinflammatory, autoimmune and neurodegenerative disorders. Harnessing this knowledge would be essential in the discovery of new molecular targets and in the development of new therapies for the aforementioned diseases.

## 5 The role of iron in CNS resident cells

As the relationship between iron and glial cells were already covered in previous reviews (46–48) [see the previous references for a detailed review on the subject], in this section, we briefly mention the role of iron in different CNS resident cell types that are relevant to MS pathophysiology and highlight the contribution of the iron dyshomeotasis in these cells as an underpinning mechanism of disease progression.

### 5.1 Iron and oligodendrocytes

Apart from their known function in brain physiology, oligodendrocytes (OLs) are the main storage sites of iron in the brain (49) when compared to other glial cells and neurons (50). On the one hand, OLs have high intracellular levels of iron and this is partially so because of the high cost of lipid biosynthesis required to produce myelin (49). On the other hand, OLs are extremely sensitive to oxidative stress due to lower levels of the antioxidant enzyme glutathione and high levels of iron (51, 52). Of note, high levels of iron is required for oligodendrocyte progenitor cells (OPCs) to differentiate (49, 53). In order to prevent oxidative stress due to increased intracellular iron and subsequent cell death by ferroptosis, OLs can export iron *via* FPN that acts in concert with ferroxidases, as evidenced by increased oxidative stress in OLs deficient for hephaestin (54). Interestingly, the crosstalk between OLs and neurons is of paramount relevance to prevent increased oxidative stress and ferroptosis in neurons, as OLs can deliver ferritin heavy chain to neurons to alleviate the oxidative stress burden and rescue neuronal death (55).

Research has shown that iron dysfunction occurs in MS and its animal models and that it is key to disease development. For instance, during the course of EAE induced by MOG immunization there is increased oxidative stress in spinal cord OLs characterized by higher levels of lipid peroxidation products, such as MDA and 4-HNE, which could be reversed by the administration of the ferroptosis inhibitor ferrostatin-1 (Fer-1) (56). Moreover, demyelination is accompanied by ferroptosis of OLs, as evidenced by increased levels of NCOA4, TfR1 and HO-1 and reduced level of hephaestin in the EAE model induced by cuprizone (57). This suggests that iron metabolism dysfunction and ferroptosis play an important role in disease progression. Furthermore, during demyelination iron is released from OLs and accumulates in the extracellular space, becoming available for the uptake by microglia and infiltrating macrophages (58). This has important implications for remyelination and further neuroinflammation/neurodegeneration, as described below.

### 5.2 Iron and neurons

It is already known that there is a crosstalk between neurons and T cells that can result in disease amelioration or progression. In this regard, it is important to pinpoint that neurons induced to ferroptosis are able to secrete factors that activate T cells, specifically Th1 and Th17 cells, resulting in an aggravation of disease (29). Therefore, it is reasonable to speculate that during EAE there is intensive neuronal death due to ferroptosis (29) and that infiltrating encephalitogenic T cells may also be activated by these unknown factors secreted by neurons. Furthermore, repression of anti-ferroptosis genes (G*px4, Nfe2l2 and Gclc*, for instance) by a histone methyltransferase G9a in neurons was recently discovered as a mechanism that drives disease progression in the mouse model of MS and in humans afflicted by MS (59). Moreover, reduced level of the ferroptosis inhibitor molecule glutathione peroxidase-4 (Gpx4) in neurons was documented during the course of EAE and this was associated with increased neuronal death by ferroptosis (60). Regarding the crosstalk between iron and inflammation, previous research has shown that PICs led to increased expression of DMT1 and iron accumulation in neurons and glia (61), which potentially triggers O&NS and PICs secretion by these cells, further contributing to CNS damage.

### 5.3 Iron and astrocytes

Besides the classic function of astrocytes in brain physiology and homeostasis, they play an important role in regulating the amount of iron that enters the brain *via* the blood-brain barrier (BBB) *via* secretion of hepcidin, a molecule that antagonizes FPN in brain endothelial cells (62). Iron was shown to be essential for normal development of astrocytes (53), but iron dysfunction in astrocytes is important for neuroinflammation (46). During the course of EAE there was an increase in TrfR, DMT1, ZIP-14 and ceruloplasmin in astrocytes, but absence of ferritin (63). These results suggest that during neuroinflammation astrocytes might accumulate iron, which is in line with the fact that in the presence of PICs, the expression of FPN is downregulated, while DMT1 is increased, leading to iron retention in astrocytes (64). One possible route of this accumulated astrocytic iron is the extracellular space *via* FPN, where astrocyte-derived iron can be used by OPCs and microglia in the process of remyelination (65). This is in agreement with a study showing upregulation of astrocytic FPN after administration of the anti-inflammatory cytokine TGF-β1 (64). Interestingly, in an inflammatory environment, astrocyte-derived hepcidin was shown to trigger neuronal death due to reduced expression of FPN and, consequently, increased iron accumulation (66). Altogether, these studies that in a pro-inflammatory environment, iron accumulates in astrocytes and drives neuroinflammation and neurodegeneration. However, during homeostasis of in the presence of anti-inflammatory cytokines, this is shifted and astrocytes export iron, which can be used by microglia and OLs to drive remyelination and repair.

### 5.4 Iron and microglia

Microglia are the brain resident immune cells that play important roles in health and disease. During the course of EAE and in MS patients, increased iron content was reported in microglia/macrophages at lesion sites (63, 67). This is in agreement with high expression of TrfR in these cells (63). Interestingly, decreased expression of hephaestin and FPN and increased expression of hepdicin (a protein that promote FPN degradation) suggest these cells accumulate iron and this may contribute to disease worsening. Interestingly, it has been shown that iron overload in macrophages promotes a shift to a pro-inflammatory phenotype (68–70), which may be associated with secretion of pro-inflammatory cytokines (PICs) and metalloproteinases (MMPs), such as MMP-9, and ROS production (48), further contributing to tissue damage and disease progression. It is important to note that both anti-inflammatory and PICs can trigger increased expression of DMT1 and lowered levels of FPN in microglia (64). Moreover, iron-laden microglia were shown to have dystrophic features and may promote a second wave of iron release to the extracellular space when they die (58).

## 6 Iron metabolism modulators as a novel therapeutic approach to treat MS?

The relationship between iron and MS has long been documented. For instance, it is already known that there are iron deposits in lesions of MS patients (67, 69) and iron accumulation in the CNS during EAE (63, 71, 72). Furthermore, iron overload in the form of iron sucrose and induced i.p. was shown to cause a severe disease course and trigger the production of MDA and 4-HNE in rats (73). On the other hand, reduction of iron content was also reported in normal appearing white matter (NAWM) of patients suffering chronically with MS (58) and in marmoset induced to EAE (72). Moreover, iron deficiency has also been directly implicated in the death of dopaminergic neurons *in vivo* (74) and mice fed a diet low in iron in the form of ferrous sulfate did not develop EAE (75). Interestingly, PICs were shown to induce the expression of heme oxygenase-1 (HO-1), which promotes the release of free Fe^2+^ into the cytoplasm and subsequently an increased mitochondrial accumulation of iron in cultured glial cells (76), thus contributing to increased generation of mitochondrial ROS. This is in line with the fact that PICs trigger oxidative and nitrosative stress (O&NS) and vice-versa (77). Strikingly, iron accumulates in the brain with aging (78) and has been implicated in the development of neurodegenerative disorders, such as Alzheimer’s disease (AD) and Parkinson’s disease (PD) (79, 80).

Given the importance of this micronutrient to the pathophysiology of MS and other inflammatory and CNS disorders, the role of iron chelators in disease setting has been explored as a possible therapeutic approach (81) [for a detailed review on the subject, the reader is referred to (81)]. In this regard, deferoxamine (DFO) given orally to EAE mice since the day of immunization resulted in a less severe disease course compared to vehicle group (82). Similar effects were also reported to occur in EAE rats given DFO and this was associated with less inflammatory infiltrate in the spinal cord of DFO treated animals even when the drug was given after the disease onset and less proliferation after recall with myelin basic protein (MBP) (83). In addition, DFO lessened the disease course in SJL mice when given in the clinical phase of disease (84). All in all, iron chelators are promising therapeutic tools to tackle MS. However, since iron does not accumulate in the CNS of MS patients uniformly, caution should be exercised when using iron chelators as a therapeutic approach to treat MS, as these drugs could interfere with the generation of OPCs and the remyelination process. Furthermore, as iron plays diverse functions in the host, ranging from energy production to immunity, caution is advised when using iron chelators as unwanted side effects may emerge. It is also important to highlight that to exert a more potent therapeutic effect, especially centrally and not only in the/via the periphery, iron chelators must cross the BBB. In this regard, deferiprone is known to cross the BBB and exerts beneficial effects in the CNS (85).

## 7 Conclusion and future perspectives

**Figure 2:**
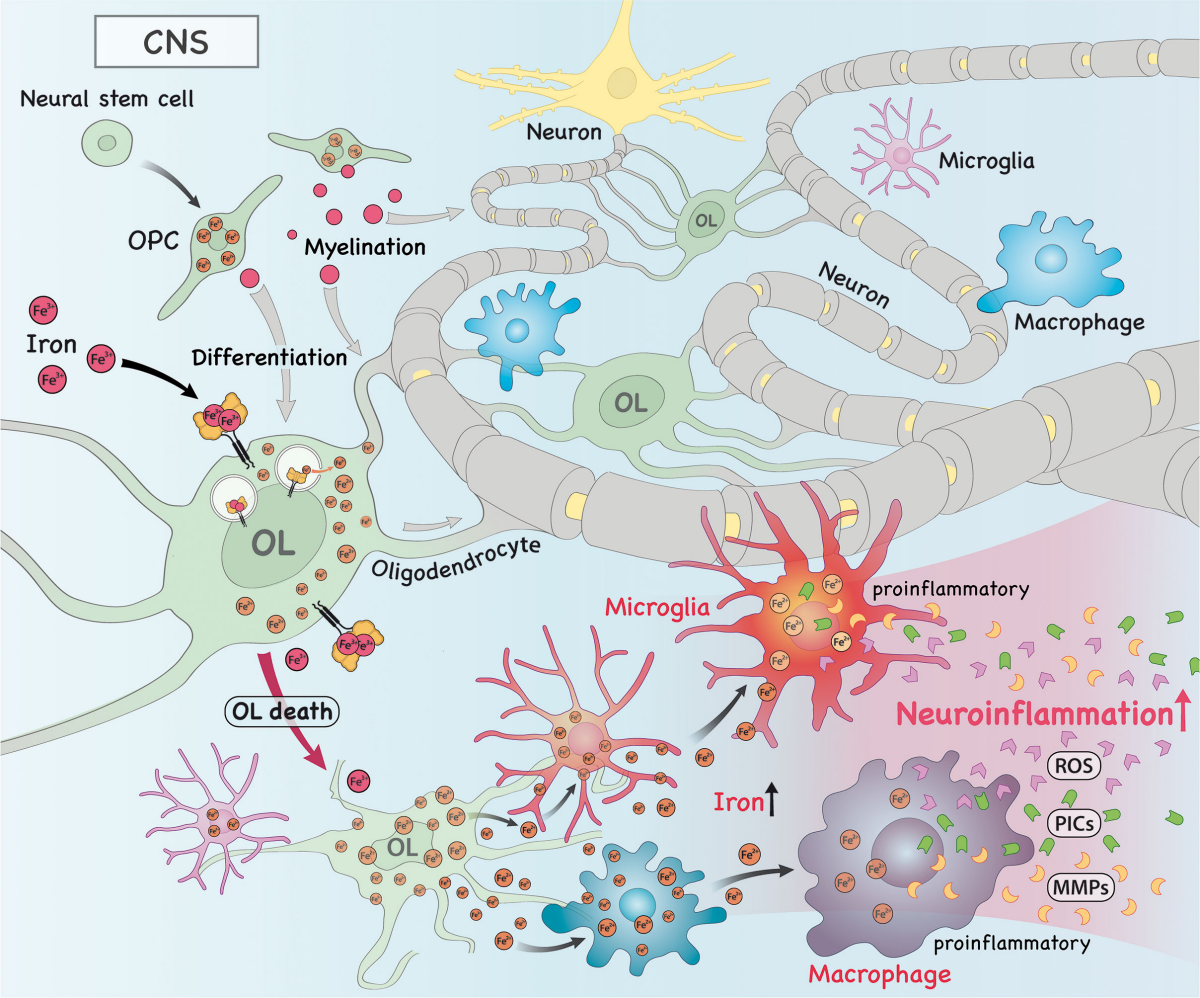
In the central nervous system (CNS), iron is imported into oligodendrocytes (OLs), which contributes to myelination and OL progenitor cell (OPC) differentiation. Alternatively, iron can also drive the death of OLs, which releases intracellular iron into the extracellular space, where it can be taken up by macrophages/microglia. As a consequence, this accumulation of iron in these cells drive them to acquire a more pro-inflammatory phenotype. These pro-inflammatory macrophages/microglia then produce and secrete high amounts of pro-inflammatory cytokines (PICs), reactive oxygen species (ROS) and metalloproteinases (MMPs), which trigger neuroinflammation.

**Figure 3:**
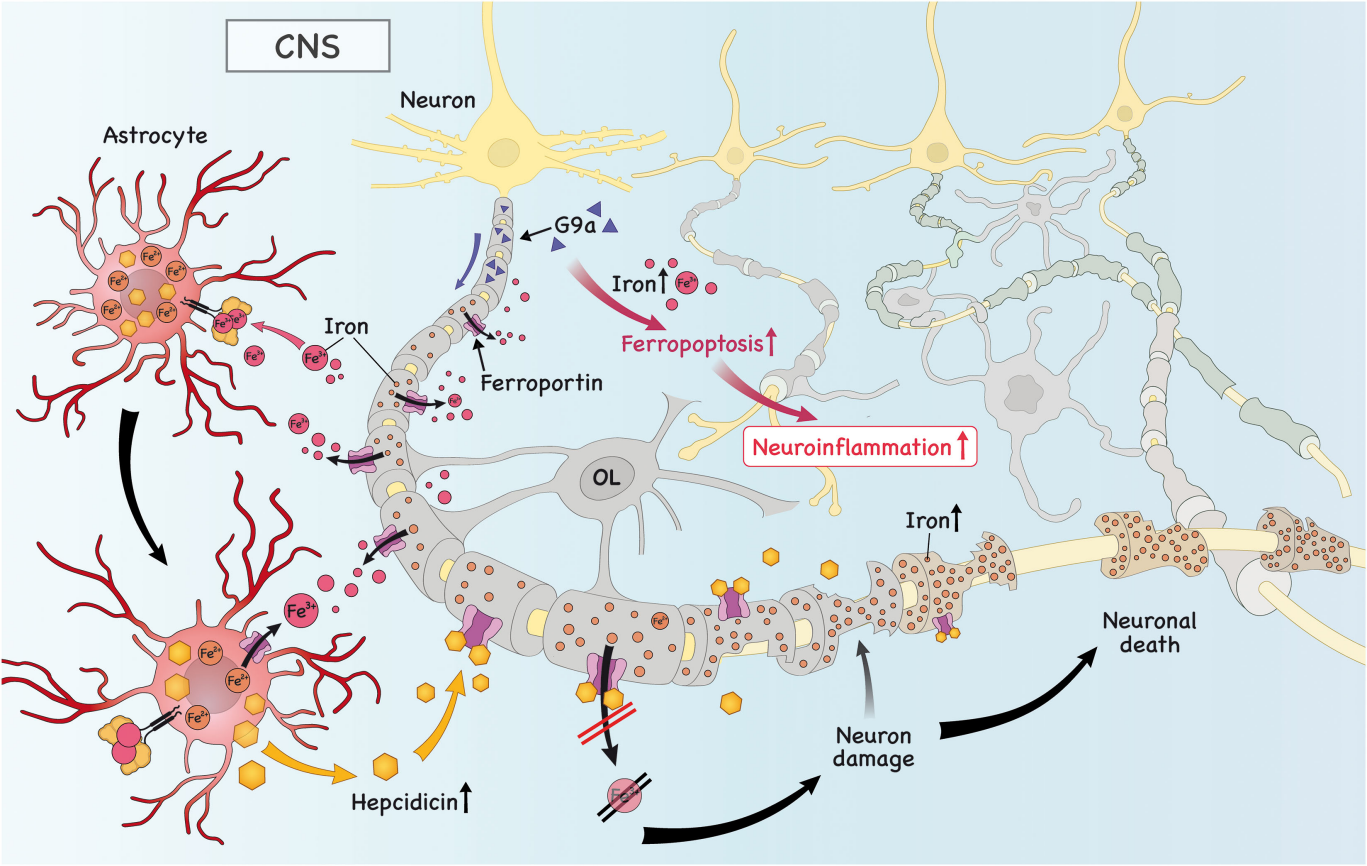
Iron is imported into astrocytes, which triggers the secretion of hepcidin. Subsequently, hepcidin binds to ferroportin (FPN) in neurons and downregulates its expression, causing iron to accumulate and neuronal death. Iron in astrocytes is exported due to high expression of astrocytic FPN to the extracellular space. There, it can be taken up by microglia/OPC, which is important to promote remyelination (not shown). Finally, the high levels of histone methyltransferase G9a in neurons promote intracellular iron accumulation, which drives ferroptosis and neuronal death, further aggravating neuroinflammation.

In conclusion, dysfunction in iron metabolism contributes to MS pathophysiology *via* a plethora of mechanisms. In addition to the mechanisms covered by a previously published paper (81), we here propose that it also aggravates MS by the generation of pathogenic T cells *via* induction of effector inflammatory cytokines, such as GM-CSF, *via* induction of a more glycolytic profile in these cells, or *via* the induction of a more pro-inflammatory profile in macrophages/microglia (**Figures 1**–**3**) and that targeting iron metabolism to inhibit the generation of such cells may be a promising therapeutic approach to treat MS and other diseases that are driven by these cells. Future studies should explore the role of iron metabolism in regulatory cells, such as regulatory innate lymphoid cells (ILCregs), Treg cells and Tr-1 cells as these are important in the regulation of an overzealous immune response and autoimmunity. These studies will likely pave the way for novel therapeutic interventions that will be key in the treatment of autoimmune disorders, such as MS.

## Author contributions

ED-S conceived the study, performed the literature search, data collection, data analysis, figure design and wrote the manuscript. CP and SM critically reviewed and edited the manuscript. All authors contributed to the article and approved the submitted version.
